# Sediment Resuspension and Deposition on Seagrass Leaves Impedes Internal Plant Aeration and Promotes Phytotoxic H_2_S Intrusion

**DOI:** 10.3389/fpls.2017.00657

**Published:** 2017-05-09

**Authors:** Kasper E. Brodersen, Kathrine J. Hammer, Verena Schrameyer, Anja Floytrup, Michael A. Rasheed, Peter J. Ralph, Michael Kühl, Ole Pedersen

**Affiliations:** ^1^Climate Change Cluster, Faculty of Science, University of Technology SydneySydney, NSW, Australia; ^2^Freshwater Biological Laboratory, Department of Biology, University of CopenhagenCopenhagen, Denmark; ^3^Centre for Tropical Water and Aquatic Ecosystem Research (TropWater), James Cook UniversityCairns, QLD, Australia; ^4^Marine Biological Section, Department of Biology, University of CopenhagenHelsingør, Denmark; ^5^School of Plant Biology, The University of Western AustraliaCrawley, WA, Australia

**Keywords:** diffusive boundary layer, dredging, H_2_S, *in situ*, microsensors, photosynthesis, seagrass, sediment

## Abstract

**HIGHLIGHTS:**

- Sedimentation of fine sediment particles onto seagrass leaves severely hampers the plants' performance in both light and darkness, due to inadequate internal plant aeration and intrusion of phytotoxic H_2_S.

Anthropogenic activities leading to sediment re-suspension can have adverse effects on adjacent seagrass meadows, owing to reduced light availability and the settling of suspended particles onto seagrass leaves potentially impeding gas exchange with the surrounding water. We used microsensors to determine O_2_ fluxes and diffusive boundary layer (DBL) thickness on leaves of the seagrass *Zostera muelleri* with and without fine sediment particles, and combined these laboratory measurements with *in situ* microsensor measurements of tissue O_2_ and H_2_S concentrations. Net photosynthesis rates in leaves with fine sediment particles were down to ~20% of controls without particles, and the compensation photon irradiance increased from a span of 20–53 to 109–145 μmol photons m^−2^ s^−1^. An ~2.5-fold thicker DBL around leaves with fine sediment particles impeded O_2_ influx into the leaves during darkness. *In situ* leaf meristematic O_2_ concentrations of plants exposed to fine sediment particles were lower than in control plants and exhibited long time periods of complete meristematic anoxia during night-time. Insufficient internal aeration resulted in H_2_S intrusion into the leaf meristematic tissues when exposed to sediment resuspension even at relatively high night-time water-column O_2_ concentrations. Fine sediment particles that settle on seagrass leaves thus negatively affect internal tissue aeration and thereby the plants' resilience against H_2_S intrusion.

## Introduction

Anthropogenic activities in coastal waters such as dredging, point-source outfall discharges and runoff from agricultural and urban catchments lead to addition and resuspension of fine particulate material that can have substantial negative impacts on the health and fitness of seagrasses (Erftemeijer and Lewis, 2006 and references herein; York et al., 2015; Chartrand et al., 2016). Dredging operations e.g., during harbor expansion or construction work can result in direct removal of plant material and plant burial by suspended sediment. The indirect effects associated with turbid sediment plumes, have largely been attributed to reduced light availability impeding seagrass photosynthesis (e.g., Erftemeijer and Lewis, 2006; York et al., 2015). Dredging-induced seagrass mortality depends on the nature of the dredging operations including the duration and intensity (Erftemeijer and Lewis, 2006; York et al., 2015), but some larger dredging activities result in widespread sediment plumes that can significantly reduce light transmission through the water-column (Cutroneo et al., 2013). Sediment resuspension for prolonged time periods can strongly affect plant fitness. Even small reductions in light availability can cause pronounced declines in the distribution and growth of seagrass meadows (Ralph et al., 2007; Chartrand et al., 2016). Seagrasses generally have high light requirements and are therefore typically found in waters, where at least 10% of incident solar irradiance reaches the seagrass leaf canopy (Duarte, 1991).

Apart from light attenuation, sediment plumes can also result in the settling of fine sediment particles on seagrass leaves, especially if the plants are already covered by epiphytes that have high potential to trap the sediment e.g., due to their excretion of exopolymers (Pereira et al., 2009; Hamisi et al., 2013). The effects of such sediment coverage on the performance of seagrasses remain largely unexplored although such sediment layers may result in a further substantial reduction in light availability for the underlying leaves, analogous to the adverse shading effects of leaf epiphytes (Brodersen et al., 2015a). Epiphytic microalgae on seagrass leaves have also been shown to significantly increase the thickness of the diffusive boundary layer (DBL) (Koch, 1994; Brodersen et al., 2015a), that is a thin unstirred layer of water, wherein solute and gas exchange between tissues and the surrounding water occurs by molecular diffusion, which is a slow process compared to bulk exchange of solutes/gasses (e.g., Jørgensen and Revsbech, 1985; Hurd, 2000). The transport time of O_2_ across the DBL increases with the square of the DBL thickness, i.e., the diffusion path length, and increasing DBL thickness will thus affect the O_2_ exchange of the seagrass leaf substantially (Jørgensen and Des Marias, 1990; Hurd, 2000; Larkum et al., 2003; Binzer et al., 2005). During the day, thick DBLs may result in increased photorespiration due to tissue accumulation of O_2_ (e.g., Maberly, 2014), but thick DBLs can be particularly problematic during darkness, where seagrasses completely rely on the diffusive supply of O_2_ from the surrounding water-column to maintain aerobic respiration of their leaves and below-ground tissues (Borum et al., 2006; Pedersen et al., 2016).

Sediment resuspension may also result in decreased water-column O_2_ concentrations due to (i) chemical oxidation of reduced metabolites and metals (Erftemeijer and Lewis, 2006), or (ii) increased aerobic mineralization of labile organic matter accumulated in the sediment under anoxic conditions. The chemical and biological O_2_ demand of suspended particles adds to the substantial O_2_ consumption by dense seagrass meadows during night-time, potentially resulting in water-column hypoxia (Greve et al., 2003; Borum et al., 2005, 2006). Night-time water-column hypoxia can result in inadequate internal aeration of belowground seagrass tissues resulting in shrinking or disappearance of the oxic micro-shield generated by radial O_2_ loss (ROL) in the rhizosphere (Koren et al., 2015; Brodersen et al., 2015b). Decreased or absent ROL, can result in intrusion of gaseous phytotoxic H_2_S from the surrounding anoxic sediment into the plant. Once in the plant, the strong binding capacity of H_2_S with iron in cytochrome *c* oxidase in the mitochondrial respiratory electron transport chain may inhibit the seagrass metabolism and lead to increased mortality (Raven and Scrimgeour, 1997; Holmer and Bondgaard, 2001; Pérez-Pérez et al., 2012; Lamers et al., 2013). Such H_2_S intrusion into seagrasses has been demonstrated both under controlled conditions in the laboratory (Pedersen et al., 2004) and in a die-off patch in the field (Borum et al., 2005). Interestingly, seagrasses possess internal detoxification mechanisms, whereby some tissue H_2_S is oxidized to elemental sulfur within the aerenchyma (Holmer and Hasler-Sheetal, 2014; Hasler-Sheetal and Holmer, 2015). Adequate internal plant aeration is thus a perquisite for healthy seagrass meadows.

The O_2_ partial pressure (*p*O_2_) of seagrass tissues is determined by four main factors: (i) the diffusive O_2_ flux from the water-column into the leaves during darkness (Pedersen et al., 2004), (ii) photosynthetic O_2_ production during the day (Dennison, 1987; Fourqurean and Zieman, 1991), (iii) the respiratory demand of the plant that is strongly affected by the ambient temperature (Raun and Borum, 2013), and (iv) the combined sediment O_2_ demand affecting the ROL in the rhizosphere (Pedersen et al., 1998; Jensen et al., 2005; Borum et al., 2006; Frederiksen and Glud, 2006).

In the present study, we combined experimental sediment resuspension experiments with microsensor measurements to investigate (i) the rates of photosynthesis and respiration, (ii) the potential role of settled sediment particles on DBL-impedance of O_2_ exchange with the water-column, (iii) the internal O_2_ status of the meristematic tissue, and (iv) the meristematic H_2_S concentrations in the seagrass *Zostera muelleri* spp. *capricorni*. Detailed microsensor measurements were performed both under controlled laboratory conditions and *in situ*, and were coupled to the light, temperature and O_2_ conditions in the surrounding water-column. We thus tested the hypotheses that sediment deposits on seagrass leaves lead to (i) reduced photosynthetic efficiency, owing to reduced light availability, as well as reduced gas exchange with the surrounding water column, (ii) reduced internal aeration of below-ground seagrass tissue, and (iii) intrusion of H_2_S into the seagrass. Our data add important ecophysiological information on the resilience/sensitivity of seagrasses to environmental disturbances linked to anthropogenic activities associated with increases in suspended sediments.

## Materials and methods

### Seagrass and sediment collection

Specimens of *Z. muelleri* spp. *capricorni* (Asch.) S.W.L. Jacobs and marine sediment were collected from shallow waters (<2 m depth) in Narrabeen Lagoon, NSW, Australia in April 22, 2015. Narrabeen Lagoon is a large (~2 km^2^), shallow intermittently closed lagoon, with a catchment area of ~55 km^2^. A plastic corer with an inner diameter of 6.3 cm was used to sample bulk sediment cores adjacent to the investigated seagrass meadow. After sampling, seagrasses and sediment were transported to the laboratory, where they were kept in constantly aerated seawater reservoirs (23°C; salinity = 29; mimicking physicochemical water-column conditions at the sampling site) prior to further investigations.

### Sediment sieving

Multiple sieves were used to obtain the fine sediment particle fraction with <63 μm grain size, henceforth referred to as silt/clay, from a sheltered area of the lagoon. After sieving, the obtained silt/clay particles and water were left undisturbed over-night in enclosed 10 L containers to allow the suspended particles to resettle. On the following day, the supernatant was carefully drained off avoiding resuspension, and the silt/clay fraction was stored in 1 L sample jars for up to 7 days until used in subsequent experiments. Furthermore, to enable differentiation between physical effects caused by the grains themselves and effects mainly driven by microbial activity within the silt/clay, some of the obtained silt/clay was sterilized by heating it to 120°C in an oven for 2 h within sealed containers to minimize evaporation.

### Laboratory measurements

#### Experimental setup

Leaf segments from 3 randomly selected *Z. muelleri* plants were positioned horizontally in a custom-made flow chamber (Brodersen et al., 2014). Within the chamber, leaf segments were fixed onto a polystyrene plate by needles. The cut ends of the investigated leaf segments were sealed with petroleum jelly prior to experiments to seal the aerenchyma from the surrounding water. A constant flow (~1 cm s^−1^) of aerated seawater (23°C, salinity = 29) was maintained in the flow chamber via a pump submerged into a seawater reservoir. The applied flow velocity of ~1 cm s^−1^ is in the lower end of field water velocities (e.g., Gambi et al., 1990; González-Ortiz et al., 2014), but does resemble water movement especially within dense seagrass meadows in closed lagoons such as the conditions in Narrabeen Lagoon. Illumination was provided by a fiber-optic tungsten halogen lamp fitted with a collimating lens (KL-2500LCD, Schott GmbH, Germany). The downwelling photon irradiance (PAR, 400–700 nm) at the leaf surface was measured with a scalar irradiance minisensor (US-SQS/L, Walz GmbH, Germany) connected to a calibrated photon irradiance meter (LI-250A, LI-COR, USA). The leaf segments were illuminated with an incident photon irradiance of 0, 75, 200, and 500 μmol photons m^−2^ s^−1^. Water-column hypoxia was obtained by continuously flushing the seawater in the supporting water reservoir with a mixture of atmospheric air and humidified nitrogen gas. The O_2_ concentration of the water reservoir was simultaneously monitored by a submerged Clark-type O_2_ microsensor (OX-10, tip diameter of 10 μm, Unisense A/S, Aarhus, Denmark; Revsbech, 1989).

#### O_2_ microsensor measurements

We used Clark-type O_2_ microsensors (OX-50, tip diameter of ~50 μm, detection limit ~0.3 μM, Unisense A/S, Aarhus, Denmark; (Revsbech, 1989)) with a fast response time (*t*_90_ < 0.5 s) and a low stirring sensitivity (<2–3%) to measure the O_2_ concentration at and toward the leaf surface. The O_2_ microsensors were mounted on a motorized micromanipulator (Unisense A/S, Aarhus, Denmark) and connected to a microsensor multimeter (Unisense A/S, Aarhus, Denmark) both interfaced with a PC running dedicated data acquisition and positioning software (SensorTrace Pro, Unisense A/S, Aarhus, Denmark). The O_2_ microsensors were linearly calibrated from signal readings in 100% air saturated seawater and anoxic seawater (by N_2_ flushing and addition of the O_2_ scavenger Na_2_SO_3_) at experimental temperature and salinity. Prior to measurements and calibrations, the microsensors were pre-conditioned with H_2_S to prevent drifting calibrations when exposed to H_2_S during experiments (Brodersen et al., 2015a). Microsensors were carefully positioned at the leaf tissue surface (defined as 0 μm distance on figures) by manually operating the micromanipulator, while observing the leaf tissue surface and microsensor tip with a boom-stand dissection microscope (AmScope, Irvine, CA, USA). When changing the downwelling photon irradiance, steady state O_2_ conditions at the leaf surface re-occurred after ~60 min (data not shown). Microprofiles of O_2_ concentration were measured in vertical increments of 100 μm, from the leaf tissue surface to 2 mm distance away (which is in the same order of magnitude as the leaf tissue thickness).

#### Photosynthesis and respiration calculations

O_2_ fluxes across the leaf tissue surfaces were calculated using Fick's first law of diffusion:

(1) $$\begin{array}{r} {\text{J}}_{{\text{O}}_{2}}=-{\text{D}}_{0}\frac{\partial \text{C}}{\partial \text{Z}} \end{array}$$

where *D*_0_ is the molecular diffusion coefficient of O_2_ in seawater at experimental temperature and salinity (2.14 × 10^−5^ cm^−2^ s^−1^; cf. tabulated physical parameters for marine systems available at www.unisense.com), and ∂*C/*∂*z* is the linear O_2_ concentration gradient in the DBL. As we introduced a physical barrier to O_2_ diffusion at the abaxial surface by fixing the leaf onto polystyrene with a low O_2_ permeability, we take the flux estimated at the adaxial side of the seagrass leaf as representative for the net flux of O_2_ across the leaf surface, i.e., *J*_*O*2, *tot*_ = *J*_*O*2, *upper-surface*_ in dark (=respiration) and light (=net photosynthesis; assuming a photosynthetic quotient of 1 mol O_2_ produced per mol CO_2_ fixed), respectively.

The calculated net photosynthesis rates (nmol O_2_ m^−2^ s^−1^) as a function of the incident photon irradiance (E; μmol photons m^−2^ s^−1^) were fitted with an exponential saturation model (Webb et al., 1974) with an added term, *R*, to account for respiration (Spilling et al., 2010):

(2) $$\begin{array}{r} {\text{P}}_{\text{n}}\left(\text{E}\right)=\;{\text{P}}_{\text{max}}\;\left(1-e{\;}^{\frac{-\alpha \text{E}}{{\text{P}}_{\text{max}}}}\right)+\text{R} \end{array}$$

This equation enables estimation of the irradiance at the onset of photosynthesis saturation as *E*_*k*_ = *P*_*max*_*/*α, where *P*_*max*_ is the maximal net photosynthesis rate and α is the initial slope of the *P*_*n*_ vs. *E* curve. The compensation photon irradiance, *E*_*C*_, was determined as the incident photon irradiance at which the leaf tissue shifted from a net O_2_ consumption to a net O_2_ production, i.e., the photon irradiance where *P*_*n*_*(E)* = 0.

#### Bulk sediment O_2_ uptake

Depth profiles of O_2_ concentration in the bulk sediment were obtained as follows. The sediment core was submerged into a ~2 L aquarium, wherein stirring and aeration of the water column was achieved via a Pasteur pipette connected to an air-pump. The surface of the sediment was determined with a boom-stand dissection microscope (AmScope, Irvine, CA, USA) and the O_2_ microsensors were carefully positioned at the sediment surface as described above. Microprofiles were performed in vertical increments of 200 μm down to 2 cm depth, i.e., below the O_2_ penetration depth. The volume specific O_2_ consumption rate of the bulk sediment, *R*_*sed*_ (μmol O_2_ m^−3^ s^−1^), was calculated as:

(3) $$\begin{array}{r} {\text{R}}_{\text{sed}}=\frac{{\text{J}}_{{\text{O}}_{2}}}{{\text{d}}_{{\text{O}}_{2}}} \end{array}$$

where *J*_*O*2_ is the O_2_ flux at the seawater/sediment interface (μmol O_2_ m^−2^ s^−1^), i.e., the diffusive oxygen uptake (DOU) of the sediment as calculated from Equation (1), and *d*_*O*2_ is the O_2_ penetration depth in the sediment (cm) as shown in Figure S1 (Supplementary Materials).

#### Potential and biological O_2_ consumption of sieved sediment

The O_2_ consumption of the fine sediment particles used in the laboratory as well as *in situ* was determined using a slightly modified approach of Pedersen et al. (2011). The O_2_ consumption was separated into total (OX_tot_) or biological (OX_bio_) O_2_ demand in order to determine the chemical O_2_ demand as OX_chem_ = OX_tot_ – OX_bio_.

The total O_2_ consumption of the sediment fraction was determined by mixing 50 mL suspended sediment (<63 μm) with 950 mL seawater with a salinity of 28. The solution was immediately transferred into 25 mL glass vials fitted with 2 glass beads to provide mixing and mounted on a rotating wheel (8 rpm) in a constant temperature bath (20.0 ± 0.5°C) (Pedersen et al., 2013). The sediment suspension was incubated for about 1 h (exact times recorded) before the O_2_ concentration was measured in each vial using a calibrated sturdy O_2_ microsensor (OX500; Unisense A/S, Denmark). Vials with seawater but without suspended sediment served as blanks enabling calculation of the O_2_ consumption as μmol O_2_ m^−3^ sediment s^−1^.

The biological O_2_ consumption was measured on a sediment suspension, which was initially purged with atmospheric air for 15 min to oxidize reduced metals and sulfide (Raun et al., 2010). After oxidation, the sediment suspension was transferred into 25 mL glass vials and treated as described above.

### *In situ* measurements

#### Experimental setup

Two patches (~1 m in diameter) of *Z. muelleri* were enclosed by custom-made transparent, floating curtains with mixing provided by submerged pumps to simulate water motion outside the enclosures (Narrabeen Lagoon, Australia). One enclosure functioned as a *control* treatment and the other enclosure as a *silt/clay* treatment. In the silt/clay treatment, 3 pulses of 375 mL silt/clay particles (see above) were added to the water column per day to mimic a dredging operation. Sediment resuspension was initiated at the beginning of the experiments (afternoon) (pulse 1), just before sunrise (pulse 2) and at midday (pulse 3). Measurements were performed on April 17, 2015 (Series 1) and repeated on April 19, 2015 (Series 2), i.e., there were 27 h difference between Series 1 and Series 2 measurements. Within the enclosures, we measured salinity, light, temperature and O_2_ in the water column during measurements of meristematic tissue O_2_ and H_2_S concentrations. A detailed description of the *in situ* measurements is given below.

#### Internal *p*O_2_ and [H_2_S] measurements

Similar data acquisition equipment and microsensor as described above were used for the field measurements of internal O_2_ partial pressure (*p*O_2_) and H_2_S concentrations ([H_2_S]) in the meristematic tissue of *Z. muelleri* over diel cycles. Internal H_2_S concentrations were measured with Clark-type H_2_S microsensors (H2S-25, tip diameter of ~25 μm, 90% response time <10 s, detection limit ~0.3 μm, Unisense A/S, Aarhus, Denmark; Jeroschewski et al., 1996; Kühl et al., 1998) that were linearly calibrated in anoxic, acidic (pH 4) Na_2_S solutions of known H_2_S concentrations (0, 50, and 100 μM). Within the enclosures, the microsensors were mounted on micromanipulators that were supported by stabilized aluminum spears at a water depth of ~1 m. The O_2_ and H_2_S microsensors were simultaneously inserted into the briefly-exposed shoot base of the target plants close to the basal leaf meristem, which was then re-buried ~2 cm into the sediment to re-establish the biogeochemical gradients (Pedersen et al., 2004). Positioning of the O_2_ microsensors was done by observing the sensor signals during insertion until a constant signal was recorded (Borum et al., 2005). The H_2_S microsensors were inserted via a similar approach, using a combination of sensor signal responses to light exposure and positioning the electrodes at approximately the same depth into the leaf meristem tissue as the O_2_ microsensors. The intra-plant O_2_ and H_2_S concentrations were measured simultaneously inside one plant in the control treatment and one plant in the silt/clay treatment, and then replicated.

#### Physical and chemical parameters of the water-column

Diel changes in ambient incident photon irradiance (continuously measured via Odyssey light loggers; Dataflow Systems, Christchurch, NZ), water-column *p*O_2_ (via O_2_ micro-optodes; OXF500PT, Pyroscience, Aachen, Germany; connected to a 4-channel Firesting meter, PyroScience, Germany), and water-column temperature (via HOBO temperature data loggers; UA-002-08, Onset Computer Corporation, Bourne, MA, USA) were recorded over ~24 h within the enclosures. All sensors were calibrated according to the manufactures instructions, mounted on a metal spear and positioned at leaf canopy height. Logging (1 Hz) by all data loggers was synchronized with the logging of microsensors used for the intra-tissue measurements.

#### *In situ* calculations

All microsensors are temperature sensitive (e.g., Kühl and Revsbech, 2001) and thus the measurements of internal *p*O_2_ and [H_2_S] obtained by the calibrated O_2_ and H_2_S microsensors were temperature corrected using the following equations (available at www.unisense.com):

(4) $$\begin{array}{r} p{\text{O}}_{2}=\;\frac{{\text{S}}_{\text{amb}-\text{Z}}}{{\text{S}}_{\text{air}}-\text{Z}}\;{\text{P}}_{0}\;e^{\text{k}\left({\text{T}}_{\text{cal}}-\;{\text{T}}_{\text{amb}}\right)} \end{array}$$

where *S*_*amb*_ is the sensor signal measured *in situ* (mV), *S*_*air*_ is the calibration signal of the sensor determined at known partial pressure and temperature (e.g., 100% air saturation; in mV), *Z* is the zero current of the sensor measured at known partial pressure and temperature (i.e., 0% air saturation; in mV), *P*_0_ is the known partial pressure used to define *S*_*air*_ (kPa), *k* is the temperature coefficient of the respective sensor (~0.02°C^−1^; exact values for individual sensors can be provided by the manufacturer, www.unisense.com), *T*_*cal*_ is the known calibration temperature (°C), and *T*_*amb*_ is the ambient temperature (°C) continuously measured *in situ*.

(5) $$\begin{array}{r} \left[{\text{H}}_{2}\text{S}\right]=\left(\text{GS}+\;{\text{S}}_{0}\right)\;e^{\text{k}\left({\text{T}}_{\text{cal}}-\;{\text{T}}_{\text{amb}}\right)} \end{array}$$

where *G* is the slope of the calibration curve that represents the sensitivity of the sensor (μmol L^−1^ mV^−1^), *S* is the signal of the sensor (mV), *S*_0_ is a constant that describes the zero current (μmol L^−1^), *k* is the temperature coefficient of the respective sensor (~0.02°C^−1^), *T*_*cal*_ is the known calibration temperature (°C), and *T*_*amb*_ is the ambient temperature (°C) continuously determined *in situ*.

These final sensor calibrations were done after the *in situ* experiments using the temperature data obtained in the respective enclosures by the submerged HOBO temperature data loggers (HOBO, Onset Computer Corporation, Bourne, MA, USA).

### Data analysis

In the following, O_2_ is quantified as μmol L^−1^ when in solution and as kPa when in gas phase. Data obtained under controlled conditions in the laboratory, i.e., O_2_ fluxes across the leaf tissue surface, are thus presented in molar concentrations and data obtained *in situ*, i.e., meristematic O_2_ concentrations and water-column O_2_ conditions are given as partial pressures. Furthermore, all laboratory measurements were performed at 40 and 100% air equilibrium, representing water-column O_2_ conditions at night- and day-time, respectively. Non-linear curve fitting was used to estimate the relationship among variables. All data fitting and analyses were performed in OriginPro (OriginPro 8, OriginLab Corporation, Northampton, MA, USA).

## Results

### Laboratory measurements

#### Sediment and silt O_2_ consumption rates

To enable comparison of sediment activity, we determined the O_2_ demand and characteristics of the added silt/clay particles (<63 μm) and the bulk sediment without seagrass biomass. The O_2_ was depleted within the upper 1.2 mm of the bulk sediment and the sediment remained anoxic with depth (Figure S1). The volume-specific O_2_ consumption rate of the bulk sediment was estimated to 374 ± 33 μmol O_2_ m^−3^ s^−1^ (Table 1). In contrast, the fine sediment particles consumed 1319 ± 6 μmol O_2_ m^−3^ s^−1^ when taking both the biological and chemical O_2_ demand into account. The biological O_2_ demand of the silt/clay particles was 1254 ± 29 μmol O_2_ m^−3^ s^−1^ resulting in a chemical O_2_ demand of 65 μmol O_2_ m^−3^ s^−1^ (Table 1). Hence, the chemical O_2_ demand of the fine sediment particles can thus most likely be neglected.

**Table 1:** **Volume specific O_2_ consumption rates of fine sediment particles (i.e., silt/clay) and bulk sediment**.

**Sediment type**	**O_2_ consumption (μmol m^−3^ s^−1^)**
Bulk, sediment (R_sed_)	374 ± 33
Fine sediment particles (Biological O_2_ demand, OX_bio_)	1254 ± 29
Fine sediment particles (Biological and chemical O_2_ demand, OX_tot_)	1319 ± 6

*Rates are mean values ± SE; n = 4. Biological O_2_ demand refers to the O_2_ consumption of fine sediment particles oxygenated via 15 min air flushing prior to measurements. Biological and chemical O_2_ demand of fine sediment particles refers to the O_2_ consumption rate of untreated, i.e., not purged with air prior to incubation, fine sediment particles. 50 mL fine sediment particles were added to 950 mL seawater*.

#### Net photosynthesis and respiration rates

**Figure 1:**
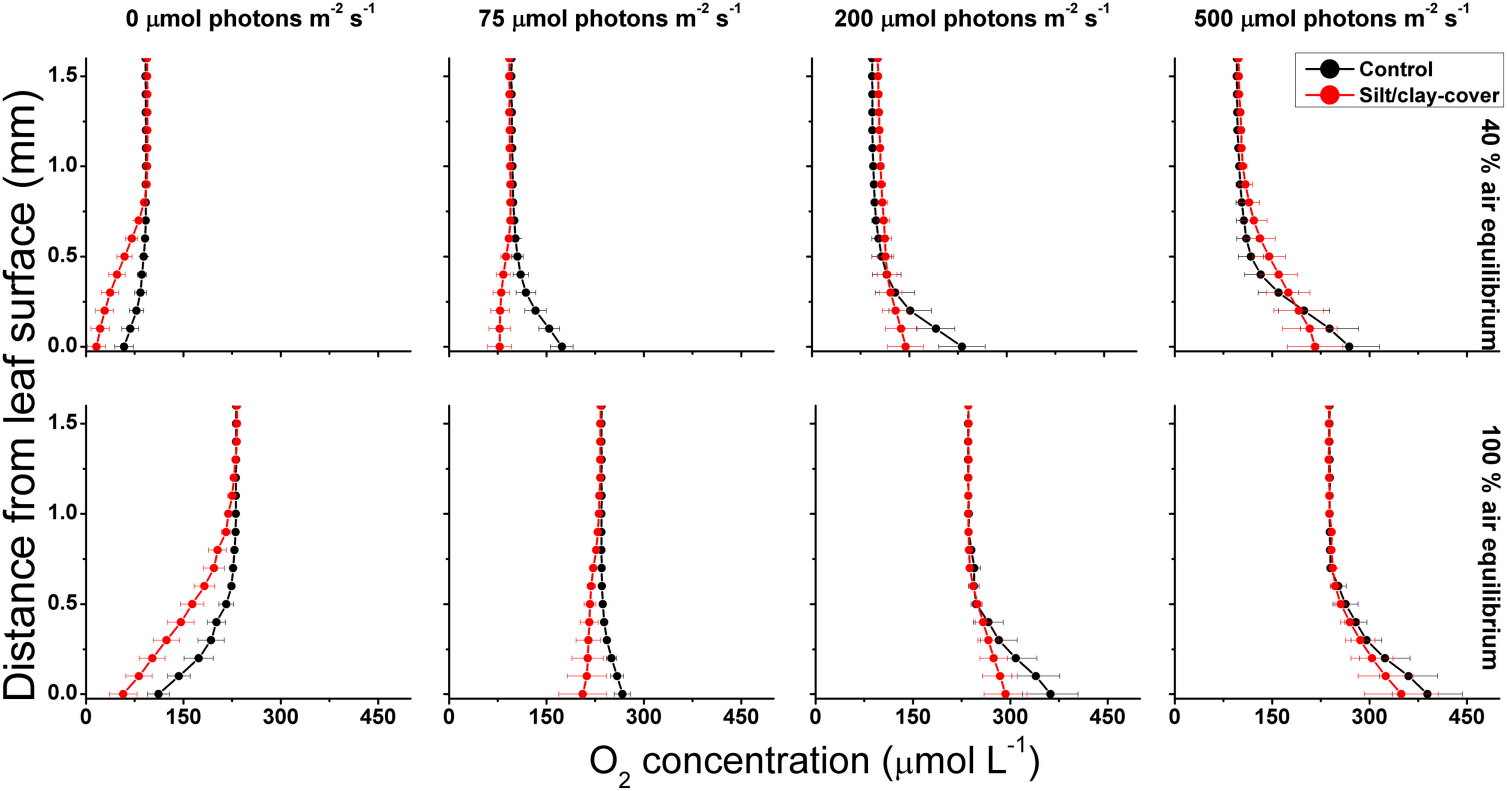
**Vertical O_2_ concentration profiles measured toward the leaf surface under incident photon irradiances of 0, 75, 200, and 500 μmol photons m^−2^ s^−1^**. Red symbols and lines represent leaves with silt/clay-cover; black symbols and lines represent control plants, i.e., leaves without silt/clay-cover. Upper panels are measurements in water with a reduced O_2_ level of ~40% of air equilibrium (mimicking night-time water-column O_2_ conditions, approximately 8.2 kPa); Lower panels are measurements in water at 100% air equilibrium (mimicking day-time water-column O_2_ conditions, 20.6 kPa). Zero depth indicates the leaf surface. Symbols and error bars represent means ± SE; *n* = 3–4.

Net photosynthesis rates increased with increasing incident photon irradiance for both plants with and without leaf silt/clay-cover (Figure 1; showing O_2_ fluxes from/into leaves). Moreover, net photosynthesis rates were higher in control leaf segments (no silt/clay added) exposed to hypoxic water conditions, resembling water-column O_2_ levels at sunrise, as compared to leaf segments kept in water at 100% air equilibrium (Table 2). Plants with leaf silt/clay-cover exhibited net O_2_ consumption already at an incident photon irradiance of ~75 μmol photons m^−2^ s^−1^ owing to reduced light availability for leaf photosynthesis (Figure 1; Table 2). Net photosynthesis rates of the control plants were 3 to 5-fold higher under moderate photon irradiance (200 μmol photons m^−2^ s^−1^) as compared to plants with leaf silt/clay-cover (Table 2). During darkness, a constant diffusive O_2_ influx across the leaf surfaces of both plants with and without leaf silt/clay-cover was observed (Figure 1). However, we found a reduction in the O_2_ flux into the silt/clay-covered leaves of 28–35% as compared to leaves without silt/clay-cover (Table 2; measured at 100% and 40% air equilibrium, respectively).

**Table 2:** **Gas exchange measured as the O_2_ flux across leaf surfaces of plants without (control)- and with fine sediment particles (<63 μm) as a function of photon irradiance**.

**Downwelling photon Irradiance (μmol photons m^−2^ s^−1^)**	**Control 40% air equilibrium (nmol O_2_ m^−2^ s^−1^)**	**With fine sediment particles 40% air equilibrium (nmol O_2_ m^−2^ s^−1^)**	**Control 100% air equilibrium (nmol O_2_ m^−2^ s^−1^)**	**With fine sediment particles 100% air equilibrium (nmol O_2_ m^−2^ s^−1^)**
0	−205 ± 57	−132 ± 3	−663 ± 223	−479 ± 44
75	435 ± 148	−18 ± 47	179 ± 61	−84 ± 143
200	854 ± 342	164 ± 110	571 ± 274	195 ± 129
500	746 ± 143	270 ± 74	701 ± 217	481 ± 266

*Positive values denote O_2_ efflux across the seagrass leaf surface. Rates are mean ± SE; n = 3–4. Note that the relative high standard errors in the silt treatment at 75 μmol photons m^−2^ s^−1^ was due to one of the leaf segments producing O_2_ via photosynthesis (for further information, please see Figure S2)*.

**Figure 2:**
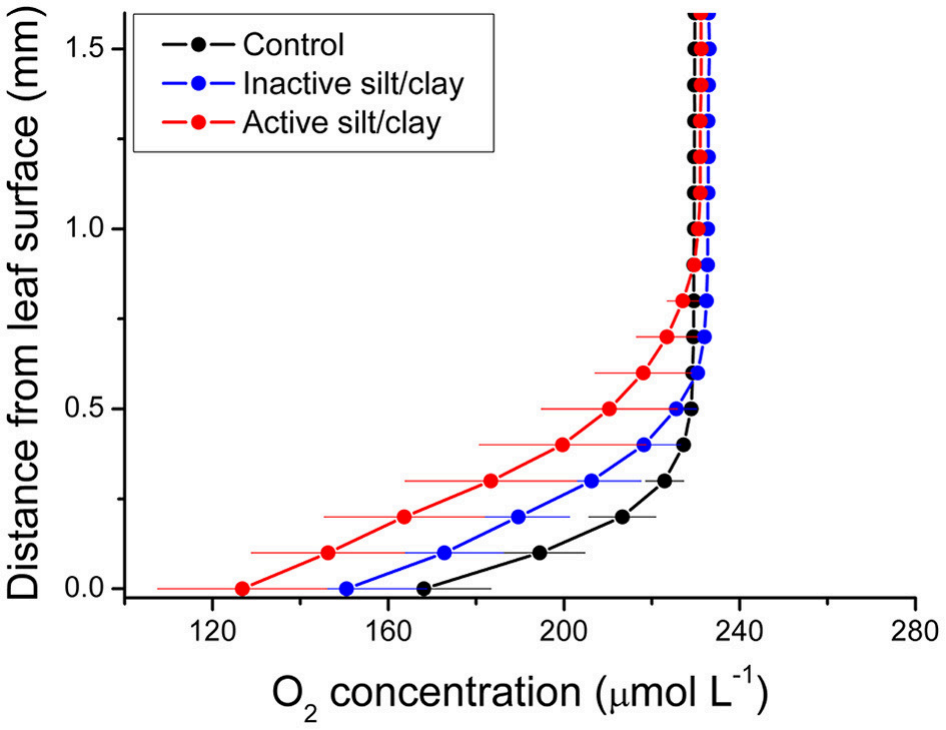
**Vertical depth profiles of the O_2_ concentration measured toward the leaf surface of plants with a microbially active silt/clay-cover (red symbols and lines), with an inactivated silt/clay-cover (obtained by pre-heating the added silt/clay to 120°C in an oven for 2 h; blue symbols and lines), and without silt/clay-cover (control plants; black symbols and lines)**. All measurements were performed in darkness. Zero depth indicates the leaf surface. The effective DBL thickness can be estimated by extrapolating the linear O_2_ concentration gradient until it intersects with the constant O_2_ concentration in the overlying water. The distance from this point into the leaf tissue surface is a measure of the effective DBL thickness (Jørgensen and Revsbech, 1985). Symbols and error bars represent means ± SE; *n* = 4.

During water-column hypoxia, the leaf silt/clay-layer impeded the diffusive O_2_ supply resulting in almost anoxic conditions at the leaf tissue surface (~16 μmol O_2_ L^−1^) of plants with leaf silt/clay-cover. This substantially increased the risk of H_2_S intrusion into the below-ground tissues during night-time as a result of inadequate internal aeration (Figure 1). The thickness of the DBL surrounding the leaves increased from ~200 μm to ~500 μm in the presence of the leaf silt/clay layer (Figure 2). This resulted in a reduction in the O_2_ influx to the leaves from 484 ± 133 nmol O_2_ m^−2^ s^−1^ in plants without leaf silt/clay-cover to 419 ± 145 nmol O_2_ m^−2^ s^−1^ in plants with an inactivated leaf silt/clay-layer. When coated with a biologically active silt/clay layer, leaves exhibited a further reduction of the O_2_ influx to 395 ± 102 nmol O_2_ m^−2^ s^−1^ (Figure 2).

**Figure 3:**
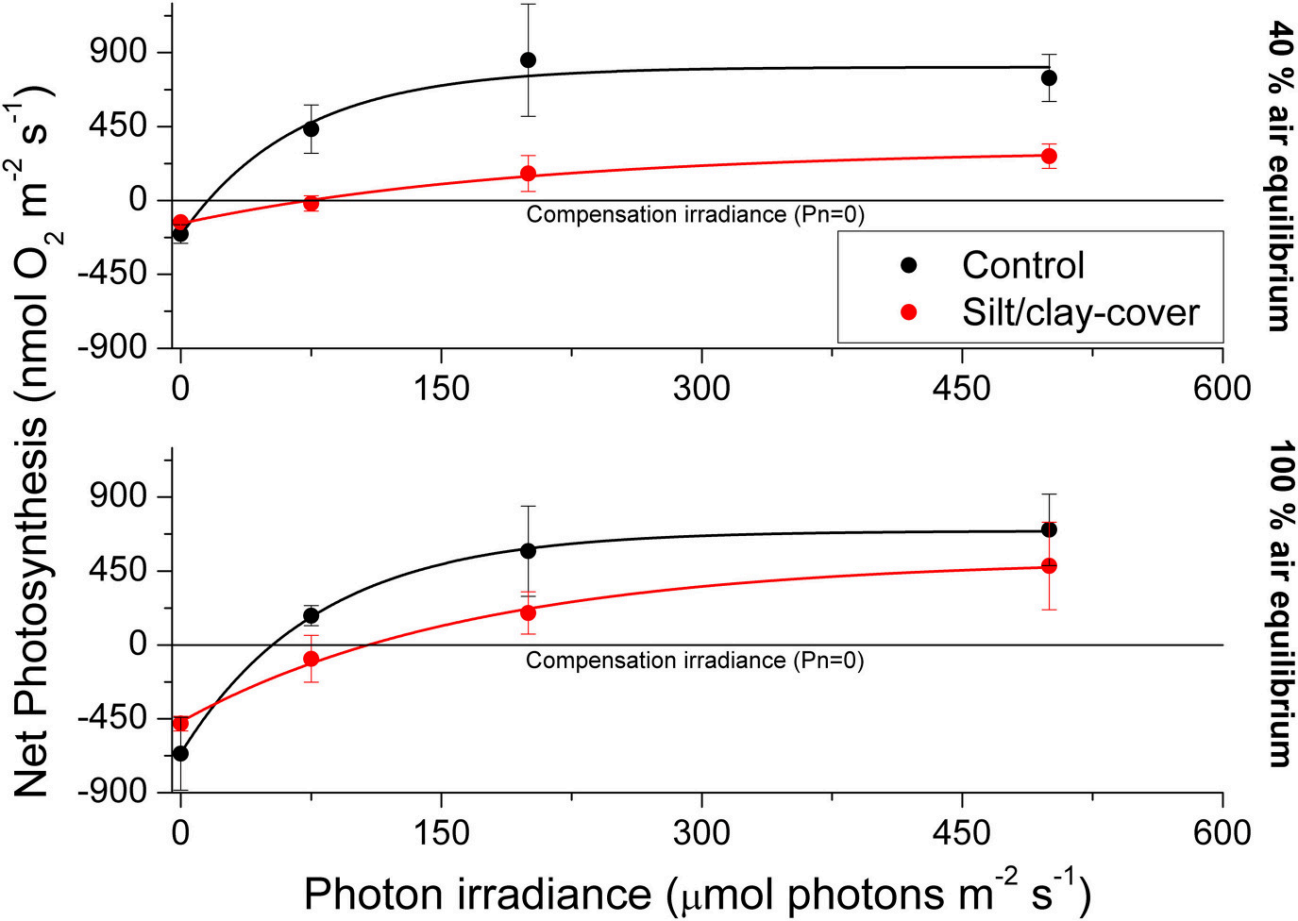
**Apparent net photosynthesis rates as a function of downwelling photon irradiance (PAR, 400–700 nm) of plants with leaf silt/clay-cover (red symbols and lines) and without leaf silt/clay-cover (control plants; black symbols and lines)**. Rates were calculated for incident photon irradiances of 0, 75, 200, and 500 μmol photons m^−2^ s^−1^ and were fitted with an exponential function (Webb et al., 1974) with an added term to account for respiration (Spilling et al., 2010) $(R_{\text{40}\%\text{ AE},\text{ control}}^{2}$ = 0.93; $R_{\text{40}\%\text{ AE},\text{ silt/clay-cover}}^{2}$ = 0.98; $R_{\text{100}\%\text{ AE},\text{ control}}^{2}$ = 0.99; $R_{\text{100}\%\text{ AE},\text{ silt/clay-cover}}^{2}$ = 0.99). The upper panel represents measurements in water kept at 40% air equilibrium, while the lower panel represents measurements in water kept at 100% air equilibrium. Error bars are ± SE; *n* = 3–4.

The silt/clay-cover on seagrass leaves resulted in a pronounced increase of the plants' compensation irradiance from 53 ± 7 μmol photons m^−2^ s^−1^ for control leaf segments to 145 ± 46 μmol photons m^−2^ s^−1^ for leaf segments with silt/clay cover, both kept in a water column at 100% air equilibrium (Figure 3; Table 3). In a water column with O_2_ kept at 40% atmospheric equilibrium, the compensation irradiance increased from 20 ± 8 μmol photons m^−2^ s^−1^ for control leaf segments to 109 ± 47 μmol photons m^−2^ s^−1^ for leaf segments with silt/clay cover (Figure 3; Table 3). The leaf silt/clay-layer effects on plant photosynthesis and respiration lead to a ~2.4-fold increase in the irradiance causing onset of net photosynthesis saturation for plants with leaf silt/clay-cover as compared to plants without leaf silt/clay-cover (Table 3), and to a 49–72% reduction of the leaf surface O_2_ concentration in darkness for plants with a leaf silt/clay-cover as compared to plants without a leaf silt/clay-cover (Table 3).

**Table 3:** **Photosynthetic parameters derived from the light response curves in Figure 3**.

	**40% of air equilibrium**		**In air equilibrium**	
	**Control**	**Fine sediment particles**	**Control**	**Fine sediment particles**
α	15 ± 4	3 ± 1	17 ± 6	6 ± 2
*P_*max*_*	1028 ± 176	503 ± 91	1354 ± 478	1010 ± 273
*R*	−211 ± 48	−141 ± 4	−662 ± 232	−468 ± 28
*E_*C*_*	20 ± 8	109 ± 47	53 ± 7	145 ± 46
*E_*k*_*	72 ± 5	174 ± 46	77 ± 2	180 ± 36
*[O_2_], dark*	59 ± 14	16 ± 14	112 ± 17	57 ± 21

**Figure 4:**
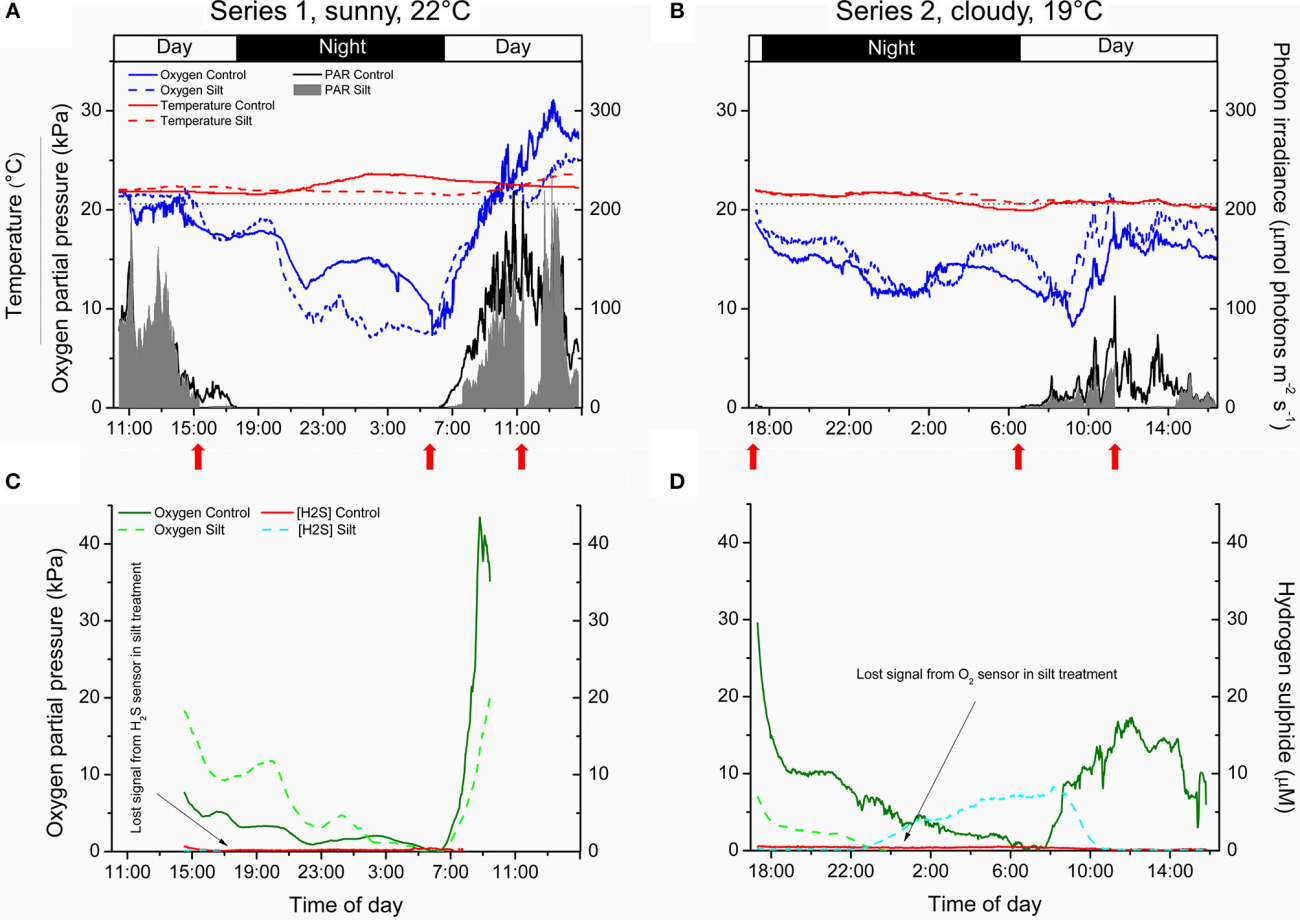
***In situ*** **measurements of diel changes in the O_2_ concentration and temperature of the water-column (A,B)**, the light availability at leaf canopy height **(A,B)**, and of the O_2_ partial pressure and H_2_S concentration in the meristematic tissue of *Zostera muelleri* plants with and without leaf silt/clay-cover, respectively **(C,D)** from Narrabeen Lagoon, NSW, Australia. The O_2_ and H_2_S microsensors were inserted into the shoot base close to the basal leaf meristem, which was buried ~2 cm into the sediment. The horizontal, dashed line in panels **(A,B)** corresponds to 100% atmospheric O_2_ partial pressure. Legends depict the physical/chemical water-column parameters **(A,B)** and the chemical species **(C,D)**. Panels **(A,C)** are from the first measuring day “Series 1” (representing a sunny day), while panels **(B,D)** are from the second measuring day “Series 2” (representing a cloudy day). Red arrows show the timing of the fine sediment pulses in the silt/clay treatment. Measurements are recorded from the exact same plants and therefore represent changes in plant performance as a result of repeated exposure to sediment re-suspension and deposition of fine sediment particles on seagrass leaves. Note the lost signal from the inserted microsensors in the silt/clay treatment **(C,D)**.

*Including photosynthetic activity, compensation irradiance, onset of photosynthesis saturation and respiration rates of investigated Zostera muelleri spp. capricorni plants with- and without (i.e., control plants) fine sediment particles on leaves. All photosynthetic related parameters were determined at both 40% of air equilibrium and in air equilibrium. n = 3. Values are mean ± SE. α = initial slope of the net photosynthesis rate vs. incident photon irradiance; P_max_ = maximum rate of net photosynthesis (in nmol O_2_ m^−2^ s^−1^); R = the respiration rate (in nmol O_2_ m^−2^ s^−1^); E_C_ = compensation irradiance (i.e., incident photon irradiance where the oxygen produced by photosynthesis meets the respiratory demands) (in μmol photons m^−2^ s^−1^); E_k_ = onset of photosynthesis saturation (in μmol photons m^−2^ s^−1^); [O_2_], dark = the leaf surface O_2_ concentration measured in darkness (in μmol L^−1^), which can be used as an estimate for the internal O_2_ concentration in the aerenchymal tissue of the thin seagrass leaves. 40% of air equilibrium mimics natural conditions in the seagrass meadow during night-time and at sunrise as seen on Figure 4. Air equilibrium mimics natural conditions during most of the day-time (Figure 4). Values are calculated/extracted from the fitted exponential saturation function (Webb et al., 1974) with an added term to account for respiration (Spilling et al., 2010) in Figure 3 (apply to: α, P_max_, R, E_c_, and E_k_) and from the O_2_ concentration microprofiles in Figure 1 ([O_2_], dark); and thus all originates from the laboratory experiments*.

### *In situ* measurements and effects of sediment re-suspension

#### Diel changes in the physical/chemical parameters of the surrounding water-column

The *p*O_2_ dynamics in the water-column of the control and silt/clay treatment showed similar patterns on a diel basis, with steadily declining *p*O_2_ during night-time reaching minimal water-column O_2_ conditions around sunrise, followed by a rapid increase in the water-column *p*O_2_ shortly after sunrise approaching atmospheric saturation (20.6 kPa) or even leading to water-column supersaturation relative to atmospheric *p*O_2_ around midday (Figures 4A,B). Water-column O_2_ levels within the enclosures fluctuated substantially during night-time owing to water bodies with varying O_2_ content being introduced to the seagrass meadow from non-vegetated areas within the lagoon and/or from the ocean due to tidal water movement. In contrast, water-column temperature remained relatively constant on a diel basis but generally decreased from ~22°C on the first measuring day (Series 1) to ~20°C at the end of the second measuring day (Series 2). Minor fluctuations in the water-column temperature during night-time correlated with the passing of aerated water bodies as observed in the water-column *p*O_2_ measurements (Figures 4A,B). The incident photon irradiance measured at leaf canopy height followed a typical bell-shaped diel curve, with minor fluctuations in the control treatment due to passing cloud cover. This was in strong contrast to the silt/clay treatment, where we measured substantially reduced light conditions as compared to the control treatment, especially in the hours following experimentally manipulated silt/clay re-suspension (Figures 4A,B). Moreover, a pronounced difference in the light availability was observed between measuring days Series 1 and Series 2, where Series 1 represented sunny conditions and Series 2 represented a cloudy late autumn day at Narrabeen Lagoon (Figures 4A,B).

#### *In situ* measurements of O_2_ and H_2_S in seagrass meristems

The internal, meristematic *p*O_2_ of both control plants and plants experimentally exposed to suspended silt/clay decreased steadily from early in the afternoon throughout the night. A minimum internal, meristematic *p*O_2_ was reached shortly after sunrise. Thereafter, a rapid increase in meristematic *p*O_2_ occurred as a response to increasing solar irradiance resulting in photosynthetic O_2_ production (Figures 4C,D). Control plants as well as silt/clay-treated plants exhibited lower *p*O_2_ relative to the water-column during night-time with tissue *p*O_2_ fluctuations correlating with changes in water-column *p*O_2_ (Figures 4A–D). A clear discrepancy in the meristematic *p*O_2_ between control plants and leaf silt/clay-treated plants was measured during light-limitation in the early morning hours (06:30–09:00) (Figure 4C) with relatively lower *p*O_2_ in silt/clay-treated plants indicating a silt/clay-induced reduction in light availability.

The meristematic below-ground tissues of both control and silt/clay-treated plants turned anoxic, or severely hypoxic, late at night. Meristematic *p*O_2_ of silt/clay-treated plants reached anoxia from around 05:00–06:30 in Series 1 and already from 23:30 in Series 2, while the control plants only were exposed to anoxic conditions in the meristematic tissue for short time periods (<1 h; Figures 4C,D). Simultaneous measurements of internal, meristematic H_2_S concentrations revealed phytotoxic H_2_S intrusion into silt/clay-treated plants during night-time in Series 2 from around 23:30 correlating with the recorded period of meristematic tissue anoxia (Figures 4C,D). Internal H_2_S levels reached a maximum of 8.3 μmol H_2_S L^−1^ around 08:00 in the morning and then started to decrease shortly after sunrise in response to photosynthetic O_2_ production leading to disappearance of H_2_S in the meristem by 10:30. No H_2_S intrusion was detected into the control plants.

#### Effects of water column O_2_ levels and silt/clay on internal O_2_ status

**Figure 5:**
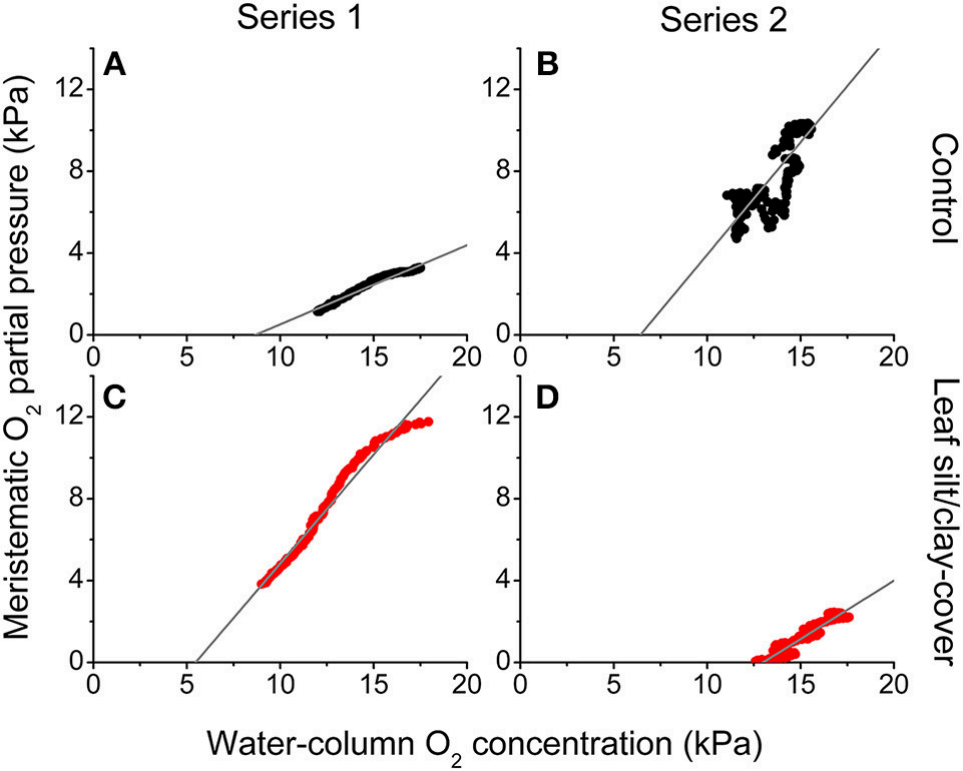
***In situ*** **intra-plant O_2_ status as a function of the O_2_ partial pressure in the surrounding water-column during night-time**. The data were extracted from Figure 4 approximately 2 h after sunset. The gray lines represent a linear regression and are extrapolated to interception with the horizontal x-axis, to provide an estimate of the water-column O_2_ level where the meristematic tissue at the shoot base becomes anoxic ($R_{\text{control},\text{ Series 1}}^{2}$ = 0.97; $R_{\text{control},\text{ Series 2}}^{2}$ = 0.70; ${R^{2}}_{\text{silt/clay-cover},\text{ Series 1}}$ = 0.97; $R_{\text{silt/clay-cover},\text{ Series 2}}^{2}$ = 0.94). Upper panels **(A,B)** are measurements from control plants (black symbols), while lower panels **(C,D)** are measurements from plants with a silt/clay-cover on the leaves (red symbols).

During night-time, tissue *p*O_2_ was derived from O_2_ in the surrounding water diffusing into the leaves and spreading via aerenchyma to below-ground tissues (Pedersen et al., 1998; Colmer, 2003; Brodersen et al., 2015a). The critical water column O_2_ level was defined as the water column *p*O_2_ below which oxic conditions in the meristematic tissue could no longer be sustained, and this critical O_2_ level was estimated by plotting the internal *p*O_2_ determined *in situ* against water-column *p*O_2_ (Figure 5). In Series 1, the meristematic tissue of the silt/clay-treated plant became anoxic at a water-column *p*O_2_ of ~5.5 kPa during night-time as compared to ~8.7 kPa in the control plant (Figures 5A,C); a tendency that dramatically changed during prolonged exposure to suspended silt/clay particles (i.e., in Series 2) where the silt/clay-treated plant became anoxic already at a night-time water-column *p*O_2_ of ~13 kPa as compared to ~6.4 kPa in the control plant (Figures 5B,D). These *in situ* findings aligned well with the lower O_2_ influx into leaves with silt/clay-cover, as compared to control leaves, determined in the controlled laboratory experiments during darkness (Figures 1–3; Tables 2,3).

**Figure 6:**
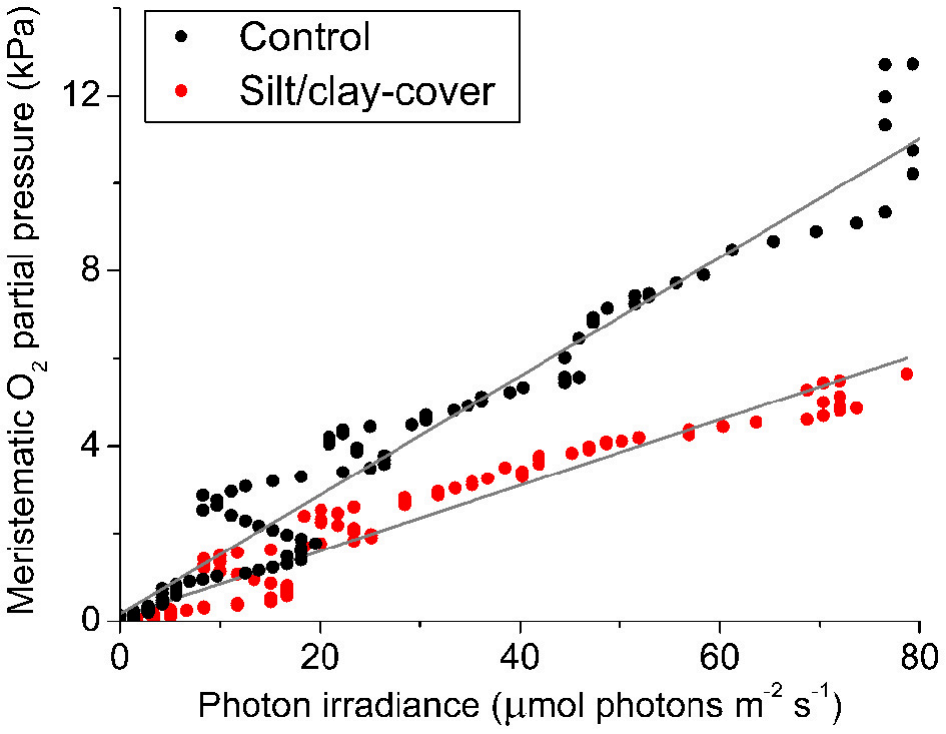
***In situ*** **intra-plant O_2_ status as a function of incident photon irradiance (PAR) during daytime**. The data were extracted from Figure 4 at sunrise (Series 1). The intra-plant O_2_ evolution during the light-limiting phase of PAR were fitted with a linear function (Gray lines) ($R_{\text{control}}^{2}$ = 0.95, α_control_ = 0.14; $R_{\text{silt/clay-cover}}^{2}$ = 0.94, α_silt/clay-*cover*_ = 0.08). Black symbols show measurements from control plants, while red symbols show measurements from plants with a silt/clay-cover on the leaves.

The silt/clay-induced shading effects on the intra-plant *p*O_2_ during natural light exposure of the seagrass leaf canopy was evaluated by plotting the *in situ* meristematic *p*O_2_ as a function of incident photon irradiance (Figure 6) revealing an ~45% reduction in meristematic *p*O_2_ in plants exposed to suspended silt/clay as compared to control plants, seen as a decrease in α, i.e., the slope describing the internal O_2_ evolution as a function of photon irradiance, from 0.14 to 0.08 (Figure 6).

## Discussion

Our results provide strong evidence that silt/clay-cover on seagrass leaves can have substantial negative effects on the plants' photosynthetic activity and efficiency, as well as on the night-time O_2_ exchange between leaf tissue and the surrounding water. Reduced internal aeration, and thus decreased below-ground tissue oxidation capacity, rendered plants with leaf silt/clay-cover more prone to H_2_S intrusion even at relatively high water-column *p*O_2_ during night-time. Below, we discuss in detail the implications of reduced light availability for photosynthesis owing to silt/clay shading, thicker DBLs, and the introduction of O_2_ consumption within the DBL itself, on internal aeration and whole plant performance of seagrasses.

### Sediment and silt/clay characteristics

We measured an ~3.4-fold higher volumetric O_2_ consumption rate of the fine sediment particles (<63 μm), as compared to the bulk sediment, indicative of high microbial activity within the thin silt/clay layer covering the leaf (Table 1). Microbial O_2_ respiration was the quantitatively most important O_2_ consuming process of the fine sediment particles, while chemical oxidation only accounted for ~5% of the total O_2_ demand (Table 1). Hence, the leaf silt/clay-cover not only impeded gas and nutrient exchange with the surrounding water-column owing to the enhanced thickness of the DBL around the leaves (Figure 2), it also reduced the passive O_2_ influx across the silt/clay layer during night-time owing to high microbial O_2_ consumption within the silt/clay layer.

### Sediment-cover effects on seagrass photosynthesis and O_2_ uptake

In light, the apparent net photosynthesis rates of *Z. muelleri* leaves with silt/clay-cover were greatly reduced as compared to control leaves, and the reduction was most pronounced at low to moderate photon irradiances (Figure 3; Table 2). The reduced leaf photosynthesis was, most likely, a combined negative result of lower light availability at the tissue surface and a DBL-impeded uptake of CO_2_ from the surrounding water-column, potentially leading to enhanced photorespiration and thereby reduced photosynthetic efficiency owing to inorganic carbon limitation (e.g., Maberly, 2014; Figures 1,3; Table 2). Consequently, the compensation irradiance of photosynthesis for plants with leaf silt/clay-cover increased to ~109 and 145 μmol photons m^−2^ s^−1^, as compared to ~20 and 53 μmol photons m^−2^ s^−1^ for control plants kept in water with O_2_ at 40% air equilibrium and 100% air equilibrium, respectively (Table 3). Silt/clay-cover can thus keep seagrass plants close to their minimum light requirements on days with poor light conditions. However, in the present experimental set-up we were unable to clearly separate the effect of reduced net photosynthesis caused by reduced light (shading by particles) or increased resistance to CO_2_ influx (thicker DBL) from that of O_2_ consumption by bacteria within the silt/clay layer.

In darkness, the passive O_2_ influx was also strongly affected by the leaf silt/clay-cover, causing a reduction of up to 35% in the O_2_ supply (Table 2), which resulted in reduced internal aeration (Table 3) especially under hypoxic water-column conditions and thus markedly increased the risk of over-night tissue anoxia. The lower O_2_ influx was a combined negative result of an increased DBL thickness impeding the exchange of O_2_ with the surrounding water-column (Figure 2) and high microbial O_2_ consumption rates within the leaf silt/clay-cover (Figure 2; Table 1). Such reduction in the meristematic *p*O_2_ lead to a reduced capability of the silt/clay-covered seagrass plant to aerate its below-ground tissue during night-time increasing the risk for phytotoxic H_2_S intrusion (Pedersen et al., 2004; Borum et al., 2005; Brodersen et al., 2015b).

Moreover, at high irradiances the silt/clay-induced impeded gas exchange with the water column may also lead to supra-optimal internal O_2_ levels during daytime, potentially resulting in oxidative stress or damage (Brodersen et al., 2015a). Similarly, elevated temperatures may lead to a CO_2_ build-up at night-time that could result in a negative feedback on respiration, cellular pH and rates of dark fixation.

### Sediment re-suspension effects on plant meristematic O_2_ and H_2_S levels

Diel changes in the meristematic O_2_ content of seagrasses were mainly driven by irradiance (Figure 4). Experimentally manipulated silt/clay re-suspension within the enclosure of the silt/clay treatment, resulted in a pronounced decrease of light availability for seagrass photosynthesis with up to 3 h of darkening measured around midday in Series 2 (Figure 4B). The diminished light conditions resulted in reduced photosynthetic O_2_ evolution and thereby reduced meristematic *p*O_2_ in *Z. muelleri* as seen at sunrise in Series 1 (Figure 4C), thus correlating with previous findings by Borum et al. (2005). The photosynthetic efficiency of *Z. muelleri* measured *in situ* was also strongly affected by the silt/clay exposure, with an almost 2-fold decrease in the net photosynthetic O_2_ evolution of plants exposed to fine sediment particles, as compared to control plants at equivalent incident photon irradiances (Figure 6), leading to reduced internal aeration and below-ground tissue oxidation capacity. This was a result of impeded gas exchange with the surrounding water-column due to a thicker DBL in the presence of a sediment cover of leaves leading to lower photosynthetic efficiencies. The *in situ* measurements thus strongly correlated with findings of a 3–5-fold higher compensation irradiance and an ~2.4-fold increase in the irradiance at onset of photosynthesis saturation in the laboratory experiments for *Z. muelleri* leaves with silt/clay-cover as compared to control leaves (Figure 3; Table 3).

Critically low meristematic *p*O_2_ and/or tissue anoxia were only measured during night-time and occurred for longer periods of time, and at higher water-column O_2_ levels, for *Z. muelleri* in the silt/clay treatment as compared to the control treatment (Figures 4, 5). This suggests reduced O_2_ supply from the leaves to the below-ground tissue of *Z. muelleri* plants exposed to fine sediment particles. The reduced meristematic *p*O_2_ was caused by (i) the leaf silt/clay-cover induced enhanced DBL thickness impeding the passive O_2_ influx into the leaves, and (ii) high rates of microbial O_2_ consumption within the leaf silt/clay layer in line with observations in the laboratory experiments (Figures 1,2). Lowest meristematic *p*O_2_ levels were recorded around sunrise, followed by a rapid increase in the meristematic O_2_ content when sunlight supported leaf photosynthesis (Figures 4C,D). Moreover, our results clearly showed that sediment re-suspension did not have substantial negative effects on the overall O_2_ conditions within the water-column (Figures 4A,B) as previously suggested (Erftemeijer and Lewis, 2006), thus underpinning the critical importance of silt/clay leaf covers.

Plants with leaf silt/clay-cover exhibited internal meristematic tissue anoxia at higher water-column *p*O_2_ levels (~45% of air equilibrium) than plants without leaf silt/clay-cover (~37% of air equilibrium), thus correlating with the lower passive O_2_ influx into leaves with silt/clay-cover during night-time determined in the laboratory experiments (Figures 1,3). The silt/clay-induced negative effect on the intra-plant O_2_ status was aggravated during prolonged exposure to fine sediment particles in the water-column (Figure 5), where the critical water-column O_2_ level for *Z. muelleri* increased to ~63% air saturation after ~54 h of exposure to experimentally manipulated silt/clay re-suspension (Figure 5). Seagrass plants with leaf silt/clay-cover were thus more vulnerable to low water-column *p*O_2_ at night-time and are exposed to an increased risk for H_2_S intrusion.

Proof of H_2_S intrusion in seagrasses has only been demonstrated *in situ* once (Borum et al., 2005) and never under conditions of such high water column *p*O_2_ as in the silt/clay-treated plants of this study, which was in strong contrast to the control treatment, where no H_2_S intrusion was detected (Figures 4C,D). Anoxic conditions in the roots, rhizome and basal leaf meristem of seagrasses lead to ceased radial O_2_ loss (ROL) from the below-ground tissue into the immediate rhizosphere and thus resulted in sediment-produced H_2_S reaching the below-ground tissue surface (Brodersen et al., 2015b). If H_2_S enters the plant e.g., via, the root apical meristems, the transport of H_2_S to the basal leaf meristem is relatively fast as it occurs via gas-phase diffusion in the aerenchyma (Pedersen et al., 2004) and this may lead to chemical asphyxiation and thereby enhanced seagrass mortality (Lamers et al., 2013). Normally, H_2_S intrusion is prevented by plant-derived ROL creating oxic sediment microniches that are sustained as long as the below-ground tissue is supported with sufficient O_2_ from the leaf canopy (Pedersen et al., 2004; Brodersen et al., 2015b, 2016). Mature regions of seagrass roots do not leak O_2_, but instead possess barriers to ROL, and thereby most likely to H_2_S intrusion, composed by Casparian-band like structures in the root endodermis (Barnabas, 1996; Enstone et al., 2003). This important anatomical cell-wall modification significantly reduces the consumption of O_2_ along the internal diffusion path and thereby ensures an effective O_2_ transport to the most distal parts of the seagrass plant (Colmer, 2003). At sunrise, photosynthetic O_2_ evolution in the leaves of the silt/clay-treated plants lead to enhanced internal meristematic *p*O_2_ and thereby re-oxidation of intruded H_2_S around 08:00–10:00 in the morning (Figure 4D), where after the H_2_S concentration remained below the detection limit.

Unfortunately, such *in situ* microsensor measurements are extremely challenging to obtain as positioning multiple microsensors simultaneously inside the tissue at the base of the shoot while “SCUBA diving” is very challenging and time consuming, and due to, e.g., sensor breakages during night-time as a result of fish foraging in the investigated seagrass meadow, as well as, time constrains such as daylight hours when positioning the sensors. Extreme changes in weather conditions did not allow us to perform additional replication. However, our results are very consistent with previous findings *in situ* and in the laboratory (e.g., Pedersen et al., 2004; Borum et al., 2005, 2006), showing H_2_S intrusion as soon as the aerenchymal tissue becomes completely anoxic, which only occurred for longer time periods in the silt/clay-treated plant during prolonged exposure to sediment re-suspension (Figures 4, 5). This clearly demonstrates that compromised photosynthesis as a result of prolonged exposure to sediment re-suspension and deposition of fine sediment particles on seagrass leaves can result in inadequate internal tissue aeration and thereby reduced below-ground tissue oxidation capacity, which leaves the plant exposed to intrusion of reduced chemical compounds such as H_2_S. The intra-plant O_2_ conditions during night-time were similar in both the control plant and silt/clay-exposed plant during Series 1, whereas this changed completely during Series 2, where the same plants showed a very different response and the silt/clay-exposed plant became completely anoxic within a few hours after sunset at high water-column *p*O_2_ simultaneously with the recording of rapid H_2_S intrusion (Figures 4, 5).

Settling of fine sediment particles onto seagrass leaves thus severely hampers the plants' performance in both light and darkness, and thereby the health of the seagrass community as a whole. Silt/clay-induced compromised photosynthesis seemed to be the most important impediment to seagrass health in our study. Dredging-induced increased water turbidity therefore represents a severe threat to seagrass communities due to its adverse effects on internal O_2_ status, and therefore can explain the often major seagrass die-off events observed during excessive dredging activities (e.g., York et al., 2015), especially if carried out during summer-time where seagrasses are more prone to tissue anoxia owing to higher respiratory needs (Staehr and Borum, 2011; Raun and Borum, 2013); thus emphasizing the need for minimizing stress-inducing dredging operations for seagrass health.

In conclusion, the present study emphasizes the importance for seagrasses to maintain protective plant-derived oxic microshields within their rhizosphere, as sediment detoxification via ROL prevents H_2_S from accumulating to very high toxic levels in the sediment and thus prevents H_2_S from reaching the tissue surface at the most vulnerable regions of the plants (Carlson et al., 1994; Brodersen et al., 2015b). Silt/clay-induced H_2_S intrusion into *Z. muelleri* seemed tightly coupled to prolonged exposure to sediment re-suspension, such as typically found during harbor dredging activities (York et al., 2015) and resulting from river plumes (Petus et al., 2014). Leaf silt/clay-covers thus impeded the plants' performance and thereby their resilience toward H_2_S intrusion. This was as a result of a combined negative plant response to the reduced light availability for photosynthesis, thicker DBLs around leaves and enhanced leaf surface microbial respiration rates, all leading to inadequate internal aeration and reduced below-ground tissue oxidation capacity (Figure 4). Turbidity-generating activities such as dredging operations in close proximity to seagrass meadows can have strong negative effects on the fitness level and health of seagrasses through multiple pathways and may lead to increased seagrass mortality.

## Author contributions

KB, OP, MK, PR, and MR designed the research. KB, OP, KH, VS, and AF conducted the experiments. KB processed the data with help from OP and KH. KB, OP, MK analyzed the data. KB wrote the manuscript with editorial help from OP, MK, PR, and MR. All authors have given approval to the final version of the manuscript.

### Conflict of interest statement

The authors declare that the research was conducted in the absence of any commercial or financial relationships that could be construed as a potential conflict of interest.
