# Supplementary material for: Sediment Resuspension and Deposition on Seagrass Leaves Impedes Internal Plant Aeration and Promotes Phytotoxic H2S Intrusion
Source: Front Plant Sci. 2017 May 9;8:657. doi: 10.3389/fpls.2017.00657 (PMC5423392; doi:10.3389/fpls.2017.00657)
Supplement: Supplementary file 1 [file DataSheet1.DOCX]

**Sediment resuspension and deposition on seagrass leaves impedes internal plant aeration and promotes phytotoxic H_2_S intrusion**

*Running title: “Dredging effects on seagrass performance”.*

**Kasper Elgetti Brodersen^1,*^, Kathrine Jul Hammer^2^, Verena Schrameyer^1^, Anja Fløytrup^2^, Michael Rasheed^3^, Peter J. Ralph^1^, Michael Kühl^1,4^, Ole Pedersen^2,*^**

^1^Plant Functional Biology and Climate Change Cluster, Aquatic Process Group, University of Technology Sydney, Sydney, Australia

^2^Freshwater Biological Section, Department of Biology, University of Copenhagen, Copenhagen, Denmark

^3^Centre for Tropical Water & Aquatic Ecosystem Research (TropWater), James Cook University, Cairns, Australia

^4^Marine Biological Section, Department of Biology, University of Copenhagen, Helsingør, Denmark

^5^School of Plant Biology, The University of Western Australia, Crawley, Australia

^*^Corresponding authors: Kasper Elgetti Brodersen, [elgetti@hotmail.com](mailto:elgetti@hotmail.com) and Ole Pedersen, [opedersen@bio.ku.dk](mailto:opedersen@bio.ku.dk)

**FIGURE S1.** Depth microprofiles of O_2_ concentration across the water/sediment interface. Zero depth indicates the sediment surface. All microsensor measurements were performed in darkness. The investigated marine sediment originated from Narrabeen Lagoon, NSW, Australia. Symbols and error bars are mean ± SEM. n = 4.





**FIGURE S2.** Net photosynthesis rates of the three investigated *Zostera muelleri* spp*. capricorni* plants as a function of downwelling photon irradiance. Black symbols and lines represent measurements on control plants, while red symbols and lines represent measurements on plants with fine sediment particles (i.e. leaf silt/clay-cover). Left panels are measurements at 40% air saturation in the water-column (mimicking *in situ* water-column O_2_ conditions during darkness and at sunrise). Right panels are measurements in a 100% air saturated water-column (mimicking *in situ* water-column O_2_ conditions at mid-day). The O_2_ fluxes were fitted with a saturated exponential function (Webb *et al*., 1974) amended with a term, R, to account for the respiration (Spilling *et al*., 2010).
